# Clinical, Paraclinical, and Antimicrobial Resistance Features of Community-Acquired Acute Bacterial Meningitis at a Large Infectious Diseases Ward in Tehran, Iran

**Published:** 2016

**Authors:** Behrooz Heydari, Hossein Khalili, Iman Karimzadeh, Hamid Emadi-Kochak

**Affiliations:** a *Department of Clinical Pharmacy, Faculty of Pharmacy, Yazd University of Medical Sciences, Yazd, Iran. *; b *Department of Clinical Pharmacy, Faculty of Pharmacy, Tehran University of Medical Sciences, Tehran, Iran.*; c *Department of Clinical Pharmacy, Faculty of Pharmacy, Shiraz University of Medical Sciences, Shiraz, Iran.*; d *Department of Infectious Disease, Faculty of Medicine, Tehran University of Medical Sciences, Tehran, Iran.*

**Keywords:** Community acquired meningitis, Antimicrobial resistance pattern, Treatment Regimens, Clinical data

## Abstract

In this study demographic, clinical, paraclinical, microbiological, and therapeutic features of patients with community-acquired acute bacterial meningitis admitted to a referral center for infectious diseases in Iran, have been evaluated.

Medical records of adult (> 18 years) individuals with confirmed diagnosis of community-acquired bacterial meningitis during a 4-year period were retrospectively reviewed. All required data were obtained from patients’ medical charts. Available findings about antimicrobial susceptibility of isolated bacteria from CSF and/or blood were also collected. Kirby-Bauer disc diffusion method was used to determine their antimicrobial susceptibility profile. Details of medical management including antibiotic regimen, duration, patients’ outcome, and possible sequelae of meningitis were recorded.

The most commonly isolated microorganism from CSF or blood of patients was *Streptococcus pneumonia* (33.33%) followed by *Neisseria meningitidis* (27.78%) and *Haemophilus influenza *(16.67%). The most common antimicrobial regimen was ceftriaxone plus vancomycin (69.44%) followed by ceftriaxone plus vancomycin plus ampicillin (11.11%). Neurological sequelae of meningitis including cranial nerve palsy, deafness, and hemiparesis were identified in 4 (11.11%), 2 (5.56%), and 1 (2.78%) subjects, respectively. Regarding mortality, only 3 (8.33%) patients died from bacterial meningitis and the remaining 33 individuals discharged from the hospital.

In conclusion, findings of the current study demonstrated that the mean incidence of acute bacterial meningitis in a referral infectious diseases ward in Iran was 9 episodes per year. The majority cases of community-acquired acute bacterial meningitis admitted to our center had negative CSF culture and classic triad of meningitis was absent in them.

## Introduction

Bacterial meningitis is defined by an infection of the arachnoid mater as the middle part of meninges and cerebrospinal fluid (CSF) in both the subarachnoid space and the cerebral ventricles (1). It has been estimated that about 1.2 million cases of bacterial meningitis occur annually worldwide (2). In developed countries such as the United States, the estimated incidence of bacterial meningitis per year is 2.6 to 6 cases per 100,000 adults (3). This rate might be up to ten times higher in developing countries. Despite considerable advances in treatment modalities and diagnostic techniques in recent decades, bacterial meningitis remains as a challenging condition for healthcare systems with relatively high morbidity and mortality in both developed and developing countries (4). It is among the 10 most common infectious causes of death and accounts for approximately 135,000 deaths each year worldwide (5).

Meningitis epidemics or its annual incidence is not well-documented in the countries of the Middle East and North Africa. However, the World Health Organization (WHO) reports that a meningitis epidemic is widespread in Egypt, Morocco, Sudan, Saudi Arabia, and Yemen (6). In Iran, anecdotal reporting of meningitis cases began in 1982 and since 1997 each provincial university of medical science has given official and regular reports from their own area (7). For the first, Mosavi-Jarrahi *et al*. comprehensively reported a total of 4,633 meningitis cases with the case-fatality rate of 4.5% within the seven-year study period from 1996 to 2005 in the population of Tehran metropolis (8). 

Epidemiological characteristics of bacterial meningitis which largely depends to patient age, time, and geography, can provide a valuable guide and database for healthcare professionals and policy makers in optimizing relevant diagnostic, preventive, and therapeutic approaches. Unfortunately, these data are actually rare in health-care settings of our country. Therefore, we conducted the current study to determine the demographic, clinical, paraclinical, microbiological, and therapeutic features of patients with community-acquired acute bacterial meningitis admitted to a referral center for various infectious diseases in Iran.

## Experimental

*Methods*

Medical records of adult (> 18 years) individuals with confirmed diagnosis of community-acquired bacterial meningitis admitted to 60-bed infectious diseases ward of Imam Khomeini Hospital affiliated to Tehran University of Medical Sciences, Tehran, Iran during a 4-year period from 2007 to 2010 were retrospectively reviewed. The Institutional Review Board (IRB) and the Medical Ethics Committee of the hospital approved the study.

Diagnosis of community-acquired bacterial meningitis was based on relevant clinical signs and symptoms such as fever, headache, neck stiffness, or impaired consciousness in addition to at least one of the following criteria: (1) positive CSF smear on Gram or Ziehl-Neelsen staining, (2) positive CSF culture, (3) positive blood culture along with CSF neutrophilic pleocytosis (absolute neutrophils count ≥100/mm^3^) and decreased glucose level or increased protein level (4). Patients with viral, fungal, or mycobacterial meningitis were excluded. Furthermore, those with meningitis related to head trauma or neurosurgical interventions, nosocomial meningitis (defined by diagnosis more than 48 h after hospitalization or during the one week of hospital discharge) (9), and aseptic meningitis (defined by negative CSF for Gram stain or culture, or blood culture in the presence of relevant clinical features) (10) were also considered ineligible for consideration. 

All required data including demographic characteristics (age, sex), relevant clinical signs and symptoms (e.g. fever, headache, nausea and vomiting, impaired consciousness level, neck stiffness, Kernig's and Brudzinski's signs, and seizure), and related paraclinical findings (e.g. CSF smear, culture, and biochemistry, blood culture, cranial computed tomography [CT] scan) were obtained from patients’ medical charts. Available findings about antimicrobial susceptibility of isolated bacteria from CSF and/or blood were also collected. Kirby-Bauer disc diffusion method was used to determine their antimicrobial susceptibility profile. Details of medical management including antibiotic regimen, duration, patients’ outcome, and possible sequelae of meningitis were recorded. 

*Statistical analysis*

Continuous and categorical variables were expressed as means ± standard deviation (SD) and percentages, respectively. Descriptive analyses were performed by the Statistical Package for the Social Sciences (SPSS) version 15 (SPSS Inc., Chicago, IL, USA).

## Results

Thirty six patients with proven diagnosis of community-acquired acute bacterial meningitis were admitted to the ward during the 4-year period. Demographic and relevant clinical characteristics of the study population are summarized in Table 1. More than three-fifth (69.44%) of the cohorts were males. Their mean ± SD age was 35.63 ± 18.32 years. Fever (94.44%) was the most common relevant clinical symptom followed by headache (88.89%) and neck stiffness (69.44%). In contrast, the classic triad of fever, neck stiffness, and change in mental status was identified in only 14 (38.89%) patients. Predisposing factors of meningitis in our cohort were as follows: diabetes mellitus (9 cases), HIV infection (7 cases), malignancy (4 cases), chronic otitis media or sinusitis (2 cases), and alcoholism (1 case). Results of cranial CT scan at admission were available in 29 (80.56%) individuals. Among these, abnormalities were detected in 12 (41.38%) subjects including cerebral edema (5 cases), hydrocephalous (4 cases), and meningeal enhancement (3 cases).

**Table 1:** Demographic and clinical characteristics of the study population (n = 36).

**Parameter**	**N (%)**
Gender	
Male	25 (69.44)
Female	11 (30.56)
Age (years)	
18-24	11 (30.56)
25-44	12 (33.33)
45-64	4 (11.11)
≥65	3 (8.33)
Related clinical signs and symptoms	
Fever	34 (94.44)
Headache	32 (88.89)
Neck stiffness	25 (69.44)
Nausea and vomiting	24 (66.67)
Impaired mental status	21 (63.89)
Kernig	13 (36.11)
Brudzinski	9 (25)
Seizure	2 (5.56)
Cranial nerve palsy	1 (2.78)

Biochemical and hematological analyses of patients’ CSF at ward admission are given in Table 2. The predominant (41.67%) white blood cell count of CSF was between 1000- < 5000/mm^3^. The neutrophil count of majority of individuals (88.89%) was above 80 percent. CSF protein level between 100 and 500 mg/dL was detected in 47.2% subjects. Three-fourth (75%) of the cohort had CSF glucose levels less than 40 mg/dL. CSF staining and culture were positive in 46.7% and 24.3% of cases, respectively. In addition, blood culture of 14 (38.89%) individuals was positive. 

**Table 2:** Biochemical and hematologic cerebrospinal fluid analysis of patients at admission

**Variable**	**n (%)**
White blood cell count (/mm^3^)	
<100	1 (2.78)
100-<1000	2 (5.56)
1000-<5000	15 (41.67)
5000-<10000	10 (27.78)
>10000	8 (22.2)
Neutrophil count (/mm^3^)	
<20%	1 (2.78)
20-80%	3 (8.33)
>80%	32 (88.89)
Protein (mg/dL)	
<50	2 (5.56)
50-<100	6 (16.67)
100-500	17 (47.2)
>500	11 (30.56)
Glucose (mg/dL)	
<40	27 (75)
40-60	5 (13.89)
>60	4 (11.11)

The most commonly isolated microorganism from CSF or blood of patients was *Streptococcus pneumonia* (33.33%) followed by *Neisseria meningitidis* (27.78%) and *Haemophilus influenza *(16.67%) (Table 3)*.*More than three-fourth (76.54%) and half (51.45%) of *S. pneumonia *isolates were resistant to penicillin G and erythromycin, respectively. In contrast, only 12.25% *S. pneumonia *isolates were resistant to ceftriaxone. All *S. pneumonia *isolates (100%) were sensitive to vancomycin. Two out of five (40%) *N. meningitidis *isolates were non-susceptible to penicillin G. More than 95 percent (96.8%) isolates of *Staphylococcus aureus *from CSF or blood were sensitive to oxacillin. All *Klebsiella pneumonia *and *Escherichia coli *isolates were sensitive to carbapenems. 

**Table 3:** Causative pathogens isolated from cerebrospinal fluid or blood culture of the study population with positive culture (n = 18).

**Microorganism**	**n (%)**
Streptococcus pneumoniae	6 (33.33)
Neisseria meningitidis	5 (27.78)
Haemophilus influenza	3 (16.67)
Staphylococcus aureus	2 (11.11)
Klebsiella pneumonia	1 (5.56)
Escherichia coli	1 (5.56)

The antimicrobial combination regimens for management of acute bacterial meningitis were listed in Table 4. The most common antimicrobial regimen was ceftriaxone plus vancomycin (69.44%) followed by ceftriaxone plus vancomycin plus ampicillin (11.11%). The mean ± SD length of antimicrobial treatment was 13.14 ± 3.7 days (range, 7-24 days). Seven (19.44%) patients also received dexamethasone. Among these, dexamethasone was initiated about 30 min before or at the time of starting antimicrobial therapy in 5 (71.43%) individuals. The length of dexamethasone treatment varied from 3 to 5 days. 

**Table 4:** Antimicrobial regimens for treatment of proven acute bacterial meningitis in the study population.

**Antimicrobial regimen**	**n (%)**
Ceftriaxone plus vancomycin	25 (69.44)
Ceftriaxone plus vancomycin plus ampicillin	4 (11.11)
Ceftazidime plus vancomycin plus ampicillin	2 (5.56)
Cefepime plus vancomycin	2 (5.56)
Ceftriaxone	2 (5.56)
Meropenem plus vancomycin	1 (2.78)

Neurological sequelae of meningitis including cranial nerve palsy, deafness, and hemiparesis were identified in 4 (11.11%), 2 (5.56%), and 1 (2.78%) subjects, respectively. Regarding mortality, only 3 (8.33%) patients died from bacterial meningitis and the remaining 33 individuals discharged from the hospital.

## Discussion

The mean annual incidence of acute bacterial meningitis in our cohort (36/4, 9 episodes) is similar to that reported from another health-care setting in Iran (7.5 episodes per year at Ahvaz) (11) as well as other countries such as Thialand (9 episodes per year) (12) and Iceland (6 episodes per year) (13). In contrast, this rate is much higher in other centers such as Massachusetts General Hospital in the United States (19 episodes per year) (9). These discrepancies can be partially attributed to different inclusion criteria in selecting patients and defining acute bacterial meningitis. In this regards for example, Sigurdardóttir *et al.* considered both nosocomial and community-acquired bacterial meningitis in their study. However, we focused only on community-acquired bacterial meningitis (9). 

Regarding age category, majority of our cohort (63.89%) ranged between 18-44 years. This is in accordance to other epidemiological studies in Iran such as Alavi *et al.* (11), and Taghavi *et al.* (14). Our findings regarding relevant clinical signs and symptoms of acute bacterial meningitis such as fever, headache, and neck stiffness are also comparable with other epidemiological investigations from both developed and developing countries (15, 16). In addition, the rate of meningitis classic triad in the current study (38.89%) was within the range reported from other surveys (33%-66%) (9,13). Predisposing factors of meningitis identified in our cohort (*e.g*. immunocompromised state, otitis, and sinusitis) are in line with those reported from other areas such as Thailand (12), Hong Kong (17), and the Netherlands (16). 

Since CSF neutrophilic pleocytosis, decreased glucose and increased protein level were part of our definition and inclusion criteria, it is not surprising that biochemical and hematological features of patients’ CSF were predominantly in accordance to bacterial meningitis. The percentage of positive CSF culture in our cohort (24.3%) was similar to that reported from other regions in Iran such as Ahvaz (19.5%) (11) or military hospitals of Tehran and other cities (28.5%) (18). This rate is generally much higher in other countries such as Saudia Arabia (*e.g*. 65.6%) (19), Thailand (*e.g*. 60.3%) (12), United States (*e.g*. 85%) (9), and Iceland (*e.g*. 89%) (13). The high rate of negative CSF culture in our center and other settings in Iran can be explained by several issues such as inappropriate and irrational use of antimicrobial agents, overlooking technical points and items of CSF sampling, storage, or transfer to microbiological lab, and lack of adequate techniques for microbiological culturing. Furthermore, since our ward is as a tertiary health-care setting, many patients from both Tehran and other parts of Iran were referred from primary or secondary hospitals or clinics and inevitably received a number of antimicrobial agents before admission. 

Similar to reports of most relevant epidemiological studies from different countries such as Saudia Arabia (35%) (19), Egypt (42%) (20), Thailand (17%) (12), the Netherlands (51%) (16) and, the United States (71%) (21), *S. pneumonia *was the most common (33.33%) causative pathogen of acquired acute bacterial meningitis in our survey. Mehrabi-Tavana *et al.* also reported *S. pneumonia *as the leading microorganism isolated from CSF of Iranian military forces or their families with meningitis (18). More than three-fourth (76.54%) of isolated *S. pneumonia* in our cohort were resistant to penicillin. Based on our previous retrospective study regarding trend of antimicrobial resistance of gram-positive bacteria within a 4-year period in the same ward, the rate of penicillin G resistant Streptococci specimen in 2007, 2008, 2009, and 2010 were 85.71%, 76.47%, 71.43%, and 62.5%, respectively (22). In these two studies (18, 22), Kirby-Bauer disc diffusion method was used to determine antimicrobial susceptibility profile. In contrast, by exploiting data of the minimum inhibitory concentration (MIC), about 20% of pneumococci isolates in the United States were resistant to penicillin (defined as MIC ≥2 mcg/mL) (23). A review of 685 *S. pneumoniae* isolates from 14 centers in 11 Asian countries from January 2000 to June 2001 revealed that 52.4% of isolates were not susceptible to penicillin (24). Regarding the widespread emergence of penicillin-resistant pneumococcus, concurrent increasing the possibility of clinical failure with cephalosporins, and changing the definition of *S. pneumonia* resistance by the Clinical and Laboratory Standards Institute in 2008, combination of ceftriaxone or cefotaxime with vancomycin has been recommended as the first line regimen for empirical treatment of *S. pneumonia *meningitis (25-27). In line with this, the predominant antibacterial regimen in our cohort (69.44%) was ceftriaxone plus vancomycin. 

*N. meningitidis* was the second most frequent pathogen (27.78%) isolated from CSF culture of our cohort. According to results of a surveillance study on 1083 cases of bacterial meningitis in the United States between 2003 and 2007, after *S. pneumonia*, *N. meningitidis* was the second causative microorganism in adults with the frequency of 12% (21). Similar findings have been generally observed from our neighboring countries such as Saudia Arabia (19) and Kuwait (28). The prevalence of *N. meningitidis* shows a gradual decrease in recent decade in most areas. This issue was also noted by Mehrabi-Tavana *et al.* in their observational, cross-sectional study on military forces or their families from 1981 to 2010 in Iran (18). It can be partially justified by the fact that at present, all conscripts of mandatory military services (over 300,000 individuals per year) as well as Hajj pilgrims (between 800,000 to 1,000,000 individuals per year) should receive the tetravalent meningococcal polysaccharide vaccine (24-25). Currently, from the approximate 2000 new cases of meningitis report per year in our country, about 10% cases are attributed to *N. meningitidis* (7). Due to increase in the rate of penicillin resistance along with the excellent efficacy, convenient dosing, and affordability of cephalosporins, third-generation cephalosporins such as ceftriaxone are currently considered as the antimicrobial agents of choice for empirical treatment of *N. meningitidis* (25). In line with this, all our patients with positive CSF culture of *N. meningitidis* are received ceftriaxone (rather than penicillin) for management of meningitis. 

Regarding frequency, *H. influenza *took the third place among causative pathogens of acute bacterial meningitis in our cohort. Similar findings were reported by Alavi *et al.* from their center in Ahvaz, Iran (11). In a multicenter study of patients with bacterial meningitis in the United States in 1995, *H. influenza *was identified as the third most common microorganism in adults up to age 60 (29). The widespread and routine use of conjugate *H. influenzae* serotype b (Hib) vaccines in infancy has led to a dramatic decrease in *H. influenza-*related infections such as meningitis especially in children in many countries (30). However, Hib vaccination is not a mandatory part of the current immunization schedule in Iran. In contrast, in the neighboring country at Saudia Arabia, it was initiated nationally by their Ministry of Health in 2002 (31). In parallel with changes in its susceptibility profile occurred in recent years, a third-generation cephalosporin (*e.g*. ceftriaxone) is the drug of choice for treatment of *H. influenzae* meningitis (25). 

Early intravenous administration of glucocorticoids (usually dexamethasone) as an adjuvant therapy in an attempt to decrease neurologic complications of bacterial meningitis as well as mortality in adults has been the subjects of various clinical trials in both developed and developing countries with inconsistent results (32). Based on 2004 Infectious Diseases Society of America (IDSA) guidelines, in all adult patients with suspected or proven acute pneumococcal meningitis, administration of dexamethasone 15 to 20 min before or at the time of antibiotic initiation can be justifiable and beneficial in developed countries (25). In our cohort, only 5 individuals received corticosteroids according to the aforementioned recommendation. Therefore, due to limited sample size of our study, it was not feasible statistically to compare the rate of neurologic complications as well as mortality in patients received dexamethasone with those not given this adjuvant therapy. 

Neurologic complications of bacterial meningitis are not uncommon in adults with the incidence of 21-28% (9, 15). They include seizures, focal neurologic deficits (*e.g*. cranial nerve palsy, hemiparesis), and sensorineural hearing loss with the approximate frequency of 15-30% (9, 15, 16, 31), 20-50% (9, 16, 32), and 12-14% (16, 32, 33), respectively. In accordance to the relevant literature, 7 (19.44%) of our study population had some degree of neurologic sequelae at ward discharge. The mortality rate in the current survey (8.33%) is much lower than that reported from most other epidemiologic investigations (18-34%) (9, 13, 36, 37). This wide difference can be justified by the fact that we only considered mortality cases directly related to meningitis rather than total-in-hospital mortality reported from other relevant studies. 

In conclusion, findings of the current study demonstrated that the mean incidence of acute bacterial meningitis in a referral infectious diseases ward in Iran was 9 episodes per year. The majority cases of community-acquired acute bacterial meningitis admitted to our center had negative CSF culture and classic triad of meningitis was absent in them. *S. pneumonia *was identified as the most common (33.33%) causative pathogen in the present survey followed by *N. meningitidis* and *H. influenzae*. The most frequent antimicrobial combination regimen for management of community-acquired acute bacterial meningitis in our cohort was ceftriaxone plus vancomycin. Directly-related mortality rate of meningitis in our study population was 8.33% and about 20% had some degrees of neurologic sequelae at the time of ward discharge. Continued and regular surveillance of epidemiologic features as well as antimicrobial resistance pattern of causative pathogens in acute bacterial meningitis are warranted to implement different preventive measures such as identifying prognostic factors of bacterial meningitis in our population and stratifying patients in according to these criteria to provide optimal medical care and decrease its mortality and complications. 
